# Supporting elder persons in rural Japanese communities through preventive home visits by nursing students: A qualitative descriptive analysis of students' reports

**DOI:** 10.1111/phn.12596

**Published:** 2019-03-07

**Authors:** Riho Iwasaki, Kazuaki Hirai, Takayuki Kageyama, Tamae Satoh, Hiromi Fukuda, Hiromi Kai, Kiwa Makino, Kathy Magilvy, Sachiyo Murashima

**Affiliations:** ^1^ Division of Health Sciences and Nursing, Department of Community Health Nursing, Graduate School of Medicine The University of Tokyo Tokyo Japan; ^2^ The Center for Nursing Education, Research and Collaboration Oita University of Nursing and Health Sciences Oita Japan; ^3^ Faculty of Health Sciences Department of Nursing Kyoto Koka Women's University Kyoto Japan; ^4^ Department of Mental Health and Psychiatric Nursing Oita University of Nursing and Health Sciences Oita Japan; ^5^ Toka Medical Skills College Bungo‐Ohno Japan; ^6^ Department of Health and Nursing Administration Oita University of Nursing and Health Sciences Oita Japan; ^7^ Division of Nurse Practitioner Course Oita University of Nursing and Health Sciences Oita Japan; ^8^ University of Colorado College of Nursing Aurora Colorado; ^9^ Oita University of Nursing and Health Sciences Oita Japan

**Keywords:** elder person, home visit, learning, nursing student, prevention, qualitative research

## Abstract

This article describes the evaluation of an innovative nursing education curriculum project, *preventive home visiting practice,* which began full implementation in 2015, in terms of students' learning outcomes. For the purpose of learning how elder persons live in community, all the 327 undergraduate nursing students, from freshmen to seniors, provided monthly or bi‐monthly visits to home‐dwelling elderly persons aged 75 or above in their home over 1 year period, in order to provide support for their independent living and to learn from them. The students' reports submitted at the end of the first academic year were qualitatively analyzed to evaluate what they learned. They acquired multiple perspectives for understanding elder persons, including a variety of individual and environmental aspects of wellness, prevention, and community life. They also acknowledged the importance of team practice through working and collaborating with different grade levels and generations. Overall, the observed learning contents were useful for future nursing education with elder persons and facilitating critical changes in nursing education systems to address the problems of aged society.

## 1 BACKGROUND

Japan is super‐aged society with “highest life expectancy” (United Nations, 2015) facing a serious problem in terms of its aging population, particularly in rural areas. Elder persons (65 years or elder) are 27.3% in 2016 (Cabinet Office & Government of Japan, 2017), while those aged 60 or above will be 42.5% in 2050 (United Nations, 2015). For 54.7% of elder persons who need to receive care, caregivers are also families aged 65 years or above. If a person aged 75 or above needs medical service or other long‐term care service, 70%–90% of the cost for the service is paid by *the medical system for elder senior citizens* or *long‐term care insurance* in Japan. Increase in the aged population means a large social/financial burden (Ministry of Health, Labour and Welfare, 2016).

The national government has proposed *the community‐based integrated care system* which enables the elderly to keep their own way of living at home by their final days (Mitsubishi UFJ Research & Consulting, 2016). The system demands nurses an increasingly critical role to support community‐dwelling elderly to prevent daily living dysfunction, hospitalization, and institutionalization. It is also important for hospital nurses to assess requirements for an inpatient to leave hospital and to live at home. Taking above into consideration, nursing education program should include the opportunities to learn daily living of community‐dwelling elderly.

However, much current nursing education takes place in hospitals and other institutions, while students have limited opportunities to observe and care for the community‐dwelling elderly who are not immediately in need of visiting nursing care. Even in community nursing practicum, public health nurses usually bring students to the home‐dwelling elderly for episodic and time‐limited meeting, but not longitudinal or continuous one. Being faced with this situation, the need of change in nursing education for the above new role of nursing is argued (Japan Association of Nursing Programs in Universities, 2018). There is no precedent of good practice for this, although Davis and Gustafson (2015) and Pohl, Malin, and Kennell (2014) reported the efficacy of nursing students' visits to children and families in chronic health conditions.

Based on the above, Oita University of Nursing and Health Sciences (OUNHS) planned and implemented an innovative clinical practice as undergraduate program of nursing named *the preventive home visits practice*, in order to develop community‐oriented geriatric and community health nursing competencies among students. The students regularly visit community‐dwelling elderly to talk about lifestyle and health, although medical treatment or clinical care except blood pressure measurement is not conducted in the visits. The purpose of the present article is to evaluate the education curriculum in terms of students' learning outcomes.

## 2 PROGRAM DEVELOPMENT AND DESCRIPTION

### 2.1 Geographical description

The above new education program has been carried out in areas A and B surrounding OUNHS located at the top of hill. Area A, spreading on the hillside, is a suburban area including many detached houses constructed approximately 40 years ago. Its population size is 75,000, 36% of which is aged 65 or above in 2015. Many steep slopes and stairs make it difficult for frail elderly to go out. Area B, spreading to the back of the hill, is a rural area in a semi‐mountainous region. Its population size is 45,000, 42% of which is aged 65 or above in 2015. It is difficult for the residents to leave homes without cars. These areas have some clinics and rehabilitation facilities. Most of users need to go to them by car. Most of the residents in both areas are Japanese, middle‐class people.

### 2.2 Preventive home visiting practice overview

Outline of *preventive home visiting practice* was as follows. The elder residents aged 75 or above in the two areas participated in the program. A student team, consisting of a freshman, sophomore, junior, and senior, repeatedly provided monthly or bi‐monthly visits to a home‐dwelling elder person. The program has three specific aims for the students, elder participants, and community: (a) For students, to learn how the participants live in the community, and what supports their independent living. (b) For the participants, to prevent physical, mental, and social disability, and to facilitate independent living. (c) For the community, to make progress in community health, and to raise awareness as to the significance of mutual support among generations. During the visit period (approximately one hour), therefore, students talked with a participant to understand their physical, mental, and psychosocial condition and living of an elder person, considered preventive measures against disease or disability, and implemented the plans to support their health and living, adjusting the situation for each participant. For example, they talk together about the participants' personal issues (life history, family, hobby, special skills, concerning, future, etc.) and health (diet, physical exercise, daily living, pain and dysfunction, medical care, etc.). If in need, students measure blood pressure and salts in soup, cook meal, take physical exercise with participants, take a walk, or recommend a doctor. Students generally make visits on foot, by bus, or by car. They take a specially prepared home‐visit bag bearing OUNHS emblem, that includes items such as a blood‐pressure gauge, stethoscope, tape measure, stopwatch, grip strength tester, salt meter, and weight scale. Each team conducts briefing and debriefing meetings before and after the visit, in order to decide what to do on a visit, to assess their participant's situation, and to make a future plan. Each student submits a personal report in an electronic file for each visit, and also submit a final report at the end of an academic year.

To prepare the program, OUNHS began a pilot study in 2013. Based on the discussion with the stakeholders such as the self‐government associations and the local government, a small preliminary model was developed using two student teams, who provided a single home visit to two participants. In the next year, a one‐year pilot project was established prior to full implementation. Eight teams of students (*n* = 33) visited eight participants. OUNHS organized a committee for community participation in the development, implementation, and evaluation of *preventive home visits*, with members including representatives from welfare commissioners, residents' associations, social welfare councils, local medical associations, local nursing associations, community comprehensive support centers, national health insurance associations, local governments, and OUNHS faculties. By the end of 2014, OUNHS decided to revise the curriculum such that the above *preventive home visit practice* would be compulsory for all the nursing students at four grades, and the curriculum was applied to the undergraduate program in 2015.

After the preparation, we developed guidelines for *preventive home visiting practice,* as follows. Eighty participants aged 75 or above, not using skilled nursing or public welfare services, were recruited from residents' associations, social welfare commissioners, and municipal and senior centers in the two areas. Since we have 80 students a year, 80 student teams were organized, consisting of a freshman, sophomore, junior, and senior. Two or three of them visit a participant monthly or bi‐monthly, and each student provides four or more visits in a year. Since a student team continuously visit the same participant throughout multiple years as long as possible, graduating seniors are replaced by matriculating freshmen, who are then mentored by their new team members, in each academic year.

The goals for the students were, (a) to understand health and living among community ‐dwelling elderly, (b) to contribute the prevention for dysfunction among them through considering appropriate measures with them, and (c) to learn teamwork beyond academic grades. Since acquired knowledge and skills among students varies depending on the grade, we expected the goals should be different by grade. However, we did not stress the difference to the students.

All the faculties of OUNHS commits to the program, even though their major is not nursing. Each two or three faculties supervise and support two or three student teams and confirm the reports submitted by the students. However, the faculties do not accompany the students except for asking to continue the participation next year, and do not instruct what the students to do when they visit the participants except responding the students' question. If students found a new problem to be solved *in the community*, OUNHS made discussion about it with the stakeholders in the community to seek solution.

The guidelines were summarized in a handbook, and explained to students and faculties in a period of kick‐off orientation at the beginning of academic year 2015. After the orientation, they had a role playing session simulating home visits. Full implementation of *preventive home visiting practice* began in spring 2015 with 327 undergraduate nursing students visiting 80 participants. The mean age of the participants was 80.0 years.

### 2.3 Design, sample, and measures

In the present article, we report the students' experiences through this new practice, based on their reports submitted at the end of the academic year 2015. The primary question for the report was "What did you learn through the one‐year visit practice?". All the students were given the question at the end of school year, and instructed to write about their experiences narratively using a computer and upload the reports to electronic files. As a result, most of them made the reports referring to the goals of the program. We believed that descriptive data for the simple question would best demonstrate the process and outcomes, providing useful evaluation data on which to improve the project in the early phase of implementation (Sandelowski, 2000), and that open‐ended student observations were very meaningful, especially for development of further evaluation instruments. We therefore conducted a qualitative descriptive study to demonstrate students' learning outcomes one year after starting the preventive home visit practicum. Identifying information was removed from the data for the purposes of analysis; however, students were informed that the reports would be used to evaluate and improve the project. We analyzed the narrative data from the reports submitted by 327 students participating from September 2016 to August 2017.

### 2.4 Analytic strategy

Analysis of the narrative data from the student reports was conducted by four members of our research team (RI, KH, MK, and TK). First, the researchers read the reports carefully to understand their meaning. Next, we extracted portions of the reports related to learning objectives for the preventive home visit practice and coded the data, grouping the coded data into categories. Three themes were then generated by collecting common codes and comparing and examining relationships between categories. Qualitative research software (MAXQDA 12) was used to organize and assist with the analysis. RI and KH categorized and interpreted the data, the validity of which was confirmed by MK and TK through the discussion among the research team.

We used the criteria of credibility, transferability, and confirmability (Lincoln & Guba, 1985) to ensure trustworthiness and rigor of the findings. Deep descriptions were written to clarify and present the results to address credibility and transferability of findings. Further, we considered the implications of these results (transferability). We were cautious that the results might be prejudiced; therefore, the analysis was discussed with several researchers with experience in qualitative research (confirmability).

### 2.5 Ethical consideration

The study protocol was approved by the Committee on Research Ethics and Safety of OUNHS, in accordance with the Research Ethics Guideline of OUNHS and the Ethical Guidelines for Medical and Health Research Involving Human Subjects (Ministry of Health, Labour, & Welfare, 2017).

## 3 RESULTS

Three themes, “Understanding wellness and prevention”, “Understanding the life experiences in community and learning the characteristics of community”, and “Teamwork”, emerged through the qualitative descriptive analysis on the report data as below. The themes, categories, and raw data are described in detail below. Illustrative quotations are provided for a rich description of the findings.

### 3.1 Understanding wellness and prevention

For the elderly to continue to live heathy in the community, students learned the importance of “wellness” and “prevention”. Two categories emerged within this major theme: “*Importance of preventive health*” and “*Perspective of wellness*.”

#### 3.1.1 Importance of preventive health

Students described how they learned the importance of preventive health, namely preventing something bad, through the home visiting practice. One student noted the following: “I think it is important for elder persons to continue to live with pleasure. I learned that preventive commitment is important so that the elderly [do not] become sick” (Senior). As expected, the students fully grasped the idea of prevention of illness and the importance of understanding the lifestyle of community‐dwelling elderly.

#### 3.1.2 Perspective of wellness

This category described the concepts of wellness experienced by elder people living in rural community. Students' learning for this category involves the following three sub‐categories.

Students had not necessarily considered healthy individuals as the target of nursing care in previous practice areas. One noted: “Through this visiting practice, I gained a good reason to think about what kind of relationship can be had with healthy people” (Sophomore). This means she and an elder person talked together on health and living, and that the relationship was different from that she experienced in clinical settings, where she presented something, such as advice for prevention, to the elder person. In other words, they found healthy people are also targets for nursing.

Students learned that they should not only pay attention to individuals' disability (i.e., problems), but also should respect their strength. One student reflected on the following: “I learned that it is important to find the strength of elder persons and to extend them, not [only] to find out their problems. As a result, they will be able to keep their current standard of living” (Senior). As already mentioned, another student wrote: “I think it is important for elder persons to continue to live with pleasure.” (Senior).

Prior to beginning this practice, most students seemed to think that most elder persons were weak, according to their narratives. However, they learned that there were many healthy elder persons in the community through home visits, and shifted the image of elder persons. One junior student reflected on the following: “I found that community residents were living a vigorous life, even some with a disease” (Junior).

Thus, students had the opportunity to learn about the concept of wellness and prevention through involvement with healthy elder persons in home visiting practice. Furthermore, they began to learn the community in more depth.

### 3.2 Understanding the life experience in community and learning the characteristics of community

Students conducted outreach and contacted people in the community, and learned the lives of people there. Two categories emerged within the theme: “Understanding the lives of community‐dwelling elder persons” and “Learning the community.”

#### 3.2.1 Understanding the lives of community‐dwelling elder persons

Students reported that they learned the difference between the lives of inpatients and those of non‐hospitalized elder people. They found the factors such as interaction with the neighborhood that supported healthy life in the community. One student stated the following: “I could understand the usual life of the elder persons, which I could not learn within a regular hospital practice” (Sophomore).

#### 3.2.2 Learning the community

Students had strong affinity for the community where they practiced. The students firstly found what they need to understand the community. They were able to learn community issues through the comprehensive, longitudinal practice. They realized that practical knowledge about the needs in each community was essential to provide appropriate care to elder individuals living in the community, and also to the entire community. Area A was suburban area near OUNHS, while the area B was quite rural. Early in the program, freshmen students recognized basic issues of community life, as one young student noted: “I realized that it is very difficult for individuals living in an area where transportation is poor” (Freshman). Another student aptly characterized the relationship between environment and health: “I thought that active engagement in health management behaviors is very good not only for the individuals' health, but also for revitalizing the community” (Freshman). Through meeting with community members, home visits, and sometimes making wellness presentation to community members, the students began to understand the characteristics of the communities where the residents lived.

The students secondly recognized a patient as a resident living in the community. They had most often encountered patients in hospital settings, and had not known much about their everyday lives in the community. They got able to view the community from the perspective of an elder resident as a citizen rather than that of a nurse. A senior student was able to evaluate the patient as a resident in a more critical manner, stating the following: “I became able to imagine the lives of patients at home, and learned the importance of considering preventive intervention methods against health problems. I believe that this will help sustain comfort at home after discharge” (Senior). As above, the students have begun to understand the lives of individuals outside the typical nursing acute care or clinic setting. Through their observations and activities visiting people at home, they began to understand the environment surrounding the residents and affecting their health. A junior student later reported that she was able to apply the learning from home visit to the long‐stay patients she met in other clinical practicum such as psychiatric and mental health nursing.

### 3.3 Teamwork

The students reflected on the learning experiences through membership in their teams. The preventive home visit program purposefully placed the students in an assigned team that together planned, visited elder persons, and evaluated their visits. This team approach gave the students good understanding of how to cooperate with other members and the importance of teamwork in health care practice. Two categories emerged within the theme, “*Essentials for building interpersonal relationships*” and “*Necessities for those in charge of a medical care team*.”

#### 3.3.1 Essentials for building interpersonal relationships

To develop a group process in each student team, interpersonal relationships were very important. One student wrote the following: “Through my visiting practice, I had an opportunity to consider the feelings not only of the elder person, but also of my teammates” (Junior). Another noted, “It is an opportunity to consider the viewpoints of elder persons, as well as of other students (i.e., the differences between juniors and seniors)” (Junior).

#### 3.3.2 Necessities for those in charge of a medical care team

Leadership was another important lesson learned by the students through the team process. For example, one student wrote the following: “By grasping the viewpoints [of students in different grades] in comparison with my own grade level, I am able to understand how collaboration can be done sufficiently” (Junior). Meanwhile, a senior student became aware of leadership by experiencing the group process: “As a result of cooperating with teammates and fully making use of the special skills offered by each grade, I experienced the efforts necessary for team medicine” (Senior).

## 4 DISCUSSION

Home visit is an important approach of nursing (Smith, 2010), although nursing students tend to show “little understanding of a patient's life” during conventional (i.e., hospital) practice (Yamamoto, Doi, Sugimoto, Sugimoto, & Kimoto, 2012). We believe that the practice such as *the preventive home visits* can alleviate the problems in conventional nursing education, broadening the perspective to include community life, health, and wellness, even in freshmen. The students had begun to learn the importance of "wellness," "prevention," and "life" concepts relevant to community‐dwelling elderlies. This agrees with the recent review (Zhang et al., 2013) that students learn the daily life of elder persons in rural area by directly contacting the community‐dwelling elder people. The students also learned the characteristics of communities and the importance of teamwork in health care. The above learning is also similar to that Pohl et al. (2014) observed for the students who visited the children and families in chronic health conditions. As a result, each of the original aims were reflected, as illustrated by students themselves.

First of all, the students grasped the viewpoints illustrated by the category, *Perspective of wellness*. This category demonstrated that the above practice leads the students to “wellness” thinking that refers to the ways in which individuals gain a state of health. Wellness is the process of moving toward integration of human functioning, maximization of human potential (Heiss & Walden, 2010). It is important not to miss the strength related to aging, such as “including daily life pleasures” and experiences that help maintain self‐efficacy. By grasping the strength of elder persons, life‐enhancing interventions can be implemented (Yamamoto, Kodama, & Kamei, 2016).

Instead of learning these viewpoints, students are often first exposed to hospitalized patients in their practical training called *ward training* in Japan (Nozaki, 2001). This gives priority to problem‐solving thinking related to diseases, and it seems difficult for the students to view a patient as a “living person.” However, if a student assumes that age‐related changes (physical and mental) in most of the home‐dwelling elderly are due to abnormalities or problems, the orientation is unsuitable for practical training with the residents. From a perspective of lifespan development, adjustments to physical changes and changes to the social structure (i.e., retirement) are viewed as normative life events.

In future nursing education, "It is indispensable to master the ability to grasp the community.” (Japanese Nursing Association, 2015). To achieve this, our project seems to be a good first step to support future endeavors toward community nursing care, because this practice enables the students to maintain longitudinal contact with home‐dwelling elderlies, facilitating their ability to view the individuals as “living persons.” This agrees with a literature review on nursing students (Zhang et al., 2013) concluding that positive images of aging can be facilitated by increasing contact with elderlies. Student in the present study similarly seems to change their viewpoints toward aging. We therefore plan to continue this practice as basic nursing education to lead students to better understanding for the home‐dwelling elderly.

Second, previous researches show that some nursing students, even at a senior grade, are not interested in discharge support for patients, lacking awareness and understanding for community experiences related to patients' livelihood after discharge (Matsuzaki et al., 2015). However, the present results show that some students, even freshmen, could acquire the perspectives toward livelihood of the elderly, as illustrated in the category, *Understanding the lives of community‐dwelling elder persons*. This indicates a new approach that strengthen students' ability to see their patients as people with lives outside the hospital setting.

Third, the present data described how undergraduate nursing students can identify important community issues and work toward solving them, as illustrated in the category, *Learning the community*. Medical care in Japan will shift toward more community‐oriented nursing care (Iwasaki et al., 2011). Students in OUNHS will be able to learn that community nursing care is the center of healthcare to elder persons in future. Since the aged population size continues to expand (Cabinet Office & Government of Japan, 2017), nursing professionals need to be well‐versed in the community and the living environments of the elderly. Thus, establishing the knowledge in early stage of educational program is essential for nurses to promote health and well‐being among community‐dwelling elderlies. These competencies are consistent with current public/community health nursing definitions and practice (American Public Health Association, 2013; Williams, 2018). In order to present the knowledge and views to nursing students, faculty must change to obtain a community perspective (Wade & Hayes, 2010).

Finally, the effects of the present nursing practice emerged through inter‐grade or inter‐generational cooperation, as shown by the theme, *Teamwork*. In an idiom of conventional nursing education curricula, learning teamwork often means working together with classmates in the same grade/level and courses. Actual teamwork for nurses is, however, inter‐generational and multi‐occupational collaboration, as widely recommended (Feather, Carr, Garletts, & Reising, 2017; World Health Organization, 2013). The teamwork is also essential to public health and community health nursing practice. Many professionals from a variety of public health disciplines as well as nonprofessional key persons work together to serve the community. In our practice, putting students from different grade from freshman to senior into one team seemed to make the students naturally collaborate each other in the above practice. However, *the preventive home visit practice* was the first experience for all the students and faculty members. When the freshmen in the present study advance to seniors, they may find the different meaning of teamwork from that reported in the present article. This should be investigated in future.

### 4.1 Limitations and future directions

One limitation of the present study was the short time frame for evaluating the intervention efficacy. Students were assessed after only one year of implementation. Although the students' reports were excellent documents about their learning in the program, we could not confirm the meaning of ambiguous phrases. Furthermore, their perceptions and behaviors in future practical settings should be evaluated again to confirm long‐term efficiency. The evaluation also will clarify the difference in learning among grades. The results of the continuous evaluation and a long‐term follow‐up study will be reported elsewhere. It is another task to investigate how the above experiences impact later nursing educational experiences. We expect favorable effects on later experiences, as suggested by some reports shown above. We also should be careful in generalization of the present results, because they come from the data of OUNHS located in a local area.

On the other hand, the effects of the above program on faculties and participants have not been evaluated yet. As for the latter, another follow‐up study is in progress: namely, the elderly who participated in the above program will be compared with a control group in terms of aging and interest in their health. Although students found some problems to be solved in the community, such as public traffic inconvenience through the above practice, they will be reported elsewhere.

## 5 CONCLUSION

Through the qualitative descriptive study on the nursing students' reports for *the preventive home visits practice*, the favorable effects of the program on their viewpoints toward community‐dwelling elderly was demonstrated. The students acquired multiple perspectives for understanding elder persons, such as wellness, prevention, community life, and team practice. If the effects are sustainable, they are in line with societal needs. The present study may be an important step forward to strengthen community health nursing competencies and practice in Japan. We will the above challenges and report the results of more long‐term and multi‐dimensional evaluation.
